# 2,5-Dichloro-*N*-cyclo­hexyl­benzene­sulfonamide

**DOI:** 10.1107/S160053681003789X

**Published:** 2010-09-25

**Authors:** Islam Ullah Khan, Shahzad Sharif, Shumaila Batool, Ahmad Mahmood Mumtaz, Edward R. T. Tiekink

**Affiliations:** a Materials Chemistry Laboratory, Department of Chemistry, Government College, University, Lahore 54000, Pakistan; b Research & Development Drugs Wing, Ministry of Health, Islamabad 44000, Pakistan; c Department of Chemistry, University of Malaya, 50603 Kuala Lumpur, Malaysia

## Abstract

The structure of the title sulfonamide, C_12_H_15_Cl_2_NO_2_S, features a distorted tetra­hedral geometry for the S atom [maximum deviation: O—S—O = 120.23 (14)°]. One of the sulfonamide O atoms is coplanar with the benzene ring [C—C—S—O torsion angle = −174.5 (2)°], whereas the other lies well above the plane [C—C—S—O = 57.0 (3)°]. A chair conformation is found for the cyclo­hexyl ring. In the crystal, supra­molecular chains aligned along the *c* axis are formed *via* N—H⋯O hydrogen bonds; these are consolidated in the three-dimensional packing by C—H⋯O contacts involving the second sulfonamide O atom.

## Related literature

For background to the pharmacological uses of sulfonamides, see: Korolkovas (1988 ▶); Mandell & Sande (1992 ▶). For related structures, see: Khan *et al.* (2010 ▶); Sharif *et al.* (2010 ▶).
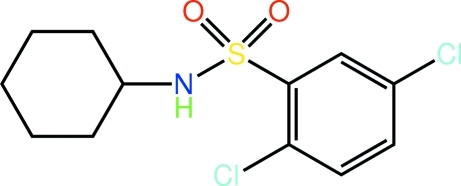


## Experimental

### 

#### Crystal data

C_12_H_15_Cl_2_NO_2_S
*M* *_r_* = 308.21
Monoclinic, 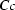

*a* = 17.4471 (12) Å
*b* = 10.7574 (8) Å
*c* = 8.2845 (6) Å
β = 111.956 (4)°
*V* = 1442.11 (18) Å^3^
*Z* = 4
Mo *K*α radiation
μ = 0.59 mm^−1^
*T* = 293 K
0.28 × 0.14 × 0.08 mm

#### Data collection

Bruker APEXII CCD diffractometer
Absorption correction: multi-scan (*SADABS*; Sheldrick, 1996 ▶) *T*_min_ = 0.692, *T*_max_ = 0.895
6491 measured reflections
2983 independent reflections
2492 reflections with *I* > 2σ(*I*)
*R*_int_ = 0.029

#### Refinement

*R*[*F* ^2^ > 2σ(*F* ^2^)] = 0.037
*wR*(*F* ^2^) = 0.092
*S* = 1.01
2983 reflections
166 parameters
3 restraints
H atoms treated by a mixture of independent and constrained refinement
Δρ_max_ = 0.24 e Å^−3^
Δρ_min_ = −0.19 e Å^−3^
Absolute structure: Flack (1983 ▶), 1327 Friedel pairs
Flack parameter: 0.06 (7)

### 

Data collection: *APEX2* (Bruker, 2007 ▶); cell refinement: *SAINT* (Bruker, 2007 ▶); data reduction: *SAINT*; program(s) used to solve structure: *SHELXS97* (Sheldrick, 2008 ▶); program(s) used to refine structure: *SHELXL97* (Sheldrick, 2008 ▶); molecular graphics: *ORTEP-3* (Farrugia, 1997 ▶) and *DIAMOND* (Brandenburg, 2006 ▶); software used to prepare material for publication: *publCIF* (Westrip, 2010 ▶).

## Supplementary Material

Crystal structure: contains datablocks global, I. DOI: 10.1107/S160053681003789X/hg2717sup1.cif

Structure factors: contains datablocks I. DOI: 10.1107/S160053681003789X/hg2717Isup2.hkl

Additional supplementary materials:  crystallographic information; 3D view; checkCIF report

## Figures and Tables

**Table 1:** Hydrogen-bond geometry (Å, °)

*D*—H⋯*A*	*D*—H	H⋯*A*	*D*⋯*A*	*D*—H⋯*A*
N1—H1*n*⋯O2^i^	0.88 (2)	2.08 (2)	2.914 (3)	157 (2)
C4—H4⋯O1^ii^	0.93	2.60	3.246 (4)	127

Symmetry codes: (i) 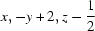; (ii) 
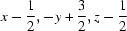
.

**Fig. 1.**
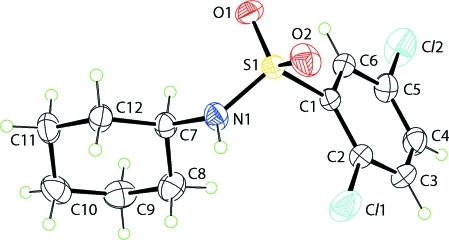
The molecular structure of (I) showing the atom-labelling scheme and displacement ellipsoids at the 50% probability level.

**Fig. 2.**
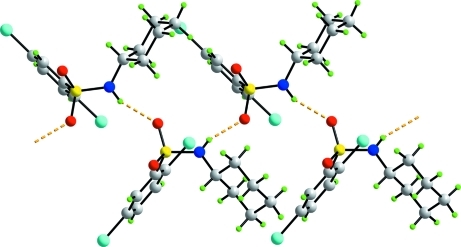
Supramolecular chain formation along c in (I) mediated by N—H···O hydrogen bonding (orange dashed lines).

**Fig. 3.**
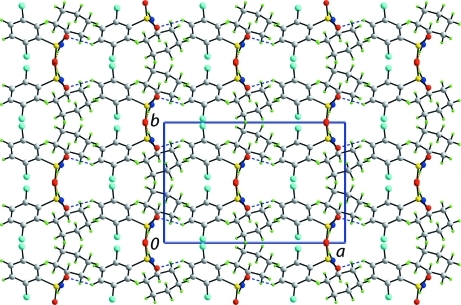
Unit-cell contents shown in projection down the c axis in (I). N—H···O hydrogen bonds (orange dashed lines) down the c axis are largely obscured. The C–H···O contacts are shown as blue dashed lines.

## supplementary crystallographic information

### Comment

Sulfonamide drugs are widely used for the treatment of certain infections caused by Gram-positive and Gram-negative microorganisms, some fungi, and certain protozoa (Korolkovas, 1988; Mandell & Sande, 1992). In continuation of on-going structural studies of sulfonamide derivatives (Khan *et al.*, 2010, Sharif *et al.*, 2010), the crystal structure of title sulfonamide, (I), is described herein.

In (I), the S atom is tetrahedrally coordinated within a CNO_2_ donor set with the greatest deviation manifested in the O1—S1—O2 angle of 120.23 (14) °. Whereas the sulfonamide-O1 atom is co-planar with the benzene ring [the O1—S1—C1—C2 torsion angle = -174.5 (2) °], the O2 atom lies well above the plane [O2—S1—C1—C2 = 57.0 (3) °]. The amide-H lies to the same side of the molecule as does the *ortho*-substituted Cl atom and approaches this atom at 2.85 (3) Å. The cyclohexyl ring adopts a chair conformation.

The presence of N1—H···O2 hydrogen bonding, Table 1, leads to the formation of supramolecular chains along the *c* axis, Fig. 2. Chains are consolidated in the 3-D packing by C4—H···O1 interactions, Fig. 3 and Table 1.

### Experimental

To 2,5-dichlorobenzenesulfonyl chloride (491 mg, 2 mmol) in 10 ml distilled water, was added cyclohexylamine (229 µl, 2 mmol) with stirring at room temperature while maintaining the pH of reaction mixture at 8 by using 3% sodium carbonate solution. The progress of reaction was monitored by TLC. After consumption of reactants, the precipitates were filtered, dried and crystallized from methanol

### Refinement

The C-bound H atoms were geometrically placed (C–H = 0.93–0.98 Å) and refined as riding with *U_iso_*(H) = 1.2*U_eq_*(C). The N-bound H atom was refined with the distance restraint N–H = 0.88±0.01 Å, and with *U_iso_*(H) = 1.2*U_eq_*(N). In the final refinement two low angle reflections evidently effected by the beam stop were omitted, *i.e*. (110) and (110).

### Crystal data

**Table:**

C_12_H_15_Cl_2_NO_2_S	*F*(000) = 640
*M**_r_* = 308.21	*D*_x_ = 1.420 Mg m^−^^3^
Monoclinic, *C**c*	Mo *K*α radiation, λ = 0.71073 Å
Hall symbol: C -2yc	Cell parameters from 2154 reflections
*a* = 17.4471 (12) Å	θ = 2.3–25.8°
*b* = 10.7574 (8) Å	µ = 0.59 mm^−^^1^
*c* = 8.2845 (6) Å	*T* = 293 K
β = 111.956 (4)°	Block, colourless
*V* = 1442.11 (18) Å^3^	0.28 × 0.14 × 0.08 mm
*Z* = 4	

### Data collection

**Table:**

Bruker APEXII CCD diffractometer	2983 independent reflections
Radiation source: fine-focus sealed tube	2492 reflections with *I* > 2σ(*I*)
graphite	*R*_int_ = 0.029
φ and ω scans	θ_max_ = 27.5°, θ_min_ = 3.8°
Absorption correction: multi-scan (*SADABS*; Sheldrick, 1996)	*h* = −22→22
*T*_min_ = 0.692, *T*_max_ = 0.895	*k* = −13→10
6491 measured reflections	*l* = −10→8

### Refinement

**Table:**

Refinement on *F*^2^	Secondary atom site location: difference Fourier map
Least-squares matrix: full	Hydrogen site location: inferred from neighbouring sites
*R*[*F*^2^ > 2σ(*F*^2^)] = 0.037	H atoms treated by a mixture of independent and constrained refinement
*wR*(*F*^2^) = 0.092	*w* = 1/[σ^2^(*F*_o_^2^) + (0.0499*P*)^2^] where *P* = (*F*_o_^2^ + 2*F*_c_^2^)/3
*S* = 1.01	(Δ/σ)_max_ = 0.001
2983 reflections	Δρ_max_ = 0.24 e Å^−^^3^
166 parameters	Δρ_min_ = −0.19 e Å^−^^3^
3 restraints	Absolute structure: Flack (1983), 1327 Friedel pairs
Primary atom site location: structure-invariant direct methods	Flack parameter: 0.06 (7)

### Special details

**Table:**

Geometry. All s.u.'s (except the s.u. in the dihedral angle between two l.s. planes) are estimated using the full covariance matrix. The cell s.u.'s are taken into account individually in the estimation of s.u.'s in distances, angles and torsion angles; correlations between s.u.'s in cell parameters are only used when they are defined by crystal symmetry. An approximate (isotropic) treatment of cell s.u.'s is used for estimating s.u.'s involving l.s. planes.
Refinement. Refinement of *F*^2^ against ALL reflections. The weighted *R*-factor *wR* and goodness of fit *S* are based on *F*^2^, conventional *R*-factors *R* are based on *F*, with *F* set to zero for negative *F*^2^. The threshold expression of *F*^2^ > 2σ(*F*^2^) is used only for calculating *R*-factors(gt) *etc*. and is not relevant to the choice of reflections for refinement. *R*-factors based on *F*^2^ are statistically about twice as large as those based on *F*, and *R*- factors based on ALL data will be even larger.

### Fractional atomic coordinates and isotropic or equivalent isotropic displacement parameters (Å^2^)

**Table:**

	*x*	*y*	*z*	*U*_iso_*/*U*_eq_	
Cl1	0.74062 (5)	0.95016 (10)	0.40157 (10)	0.0731 (3)	
Cl2	0.71411 (6)	0.53058 (11)	0.89049 (15)	0.0905 (4)	
S1	0.90528 (4)	0.86294 (6)	0.72894 (8)	0.04118 (17)	
O1	0.96062 (11)	0.7885 (2)	0.8655 (3)	0.0571 (6)	
O2	0.89603 (14)	0.99251 (19)	0.7564 (3)	0.0608 (6)	
N1	0.93057 (15)	0.8530 (2)	0.5645 (3)	0.0457 (6)	
H1n	0.9127 (18)	0.912 (2)	0.486 (3)	0.055*	
C1	0.80608 (14)	0.7947 (2)	0.6796 (3)	0.0373 (6)	
C2	0.73570 (17)	0.8342 (3)	0.5422 (4)	0.0448 (7)	
C3	0.66045 (17)	0.7810 (3)	0.5144 (4)	0.0540 (8)	
H3	0.6137	0.8089	0.4232	0.065*	
C4	0.65355 (18)	0.6871 (3)	0.6199 (4)	0.0533 (7)	
H4	0.6027	0.6499	0.5995	0.064*	
C5	0.72223 (19)	0.6489 (3)	0.7549 (4)	0.0510 (7)	
C6	0.79885 (17)	0.7007 (3)	0.7871 (3)	0.0436 (6)	
H6	0.8450	0.6729	0.8798	0.052*	
C7	0.96379 (16)	0.7403 (2)	0.5145 (4)	0.0392 (6)	
H7	0.9751	0.6793	0.6085	0.047*	
C8	0.90286 (18)	0.6836 (3)	0.3500 (4)	0.0550 (8)	
H8A	0.8874	0.7450	0.2575	0.066*	
H8B	0.8533	0.6593	0.3689	0.066*	
C9	0.9399 (2)	0.5697 (3)	0.2951 (5)	0.0608 (9)	
H9A	0.9499	0.5050	0.3821	0.073*	
H9B	0.9008	0.5379	0.1858	0.073*	
C10	1.0206 (2)	0.6020 (3)	0.2741 (4)	0.0624 (9)	
H10A	1.0442	0.5274	0.2457	0.075*	
H10B	1.0099	0.6605	0.1791	0.075*	
C11	1.0810 (2)	0.6580 (3)	0.4394 (4)	0.0564 (8)	
H11A	1.1308	0.6823	0.4216	0.068*	
H11B	1.0961	0.5961	0.5313	0.068*	
C12	1.04439 (17)	0.7717 (3)	0.4958 (4)	0.0471 (7)	
H12A	1.0834	0.8022	0.6061	0.056*	
H12B	1.0352	0.8372	0.4101	0.056*	

### Atomic displacement parameters (Å^2^)

**Table:**

	*U*^11^	*U*^22^	*U*^33^	*U*^12^	*U*^13^	*U*^23^
Cl1	0.0545 (5)	0.0915 (7)	0.0708 (6)	0.0203 (4)	0.0206 (4)	0.0393 (5)
Cl2	0.0742 (6)	0.0882 (7)	0.1198 (9)	0.0010 (5)	0.0486 (6)	0.0425 (6)
S1	0.0346 (3)	0.0513 (4)	0.0368 (3)	−0.0019 (3)	0.0124 (2)	−0.0063 (3)
O1	0.0349 (10)	0.0900 (16)	0.0400 (11)	−0.0033 (10)	0.0066 (9)	0.0081 (10)
O2	0.0674 (14)	0.0549 (12)	0.0639 (14)	−0.0091 (11)	0.0290 (11)	−0.0221 (11)
N1	0.0516 (14)	0.0457 (14)	0.0473 (14)	0.0123 (10)	0.0273 (12)	0.0108 (10)
C1	0.0318 (12)	0.0465 (15)	0.0337 (13)	0.0026 (11)	0.0122 (10)	−0.0074 (11)
C2	0.0385 (14)	0.0551 (17)	0.0391 (15)	0.0093 (12)	0.0127 (12)	0.0014 (13)
C3	0.0327 (13)	0.076 (2)	0.0446 (18)	0.0084 (15)	0.0045 (13)	−0.0017 (15)
C4	0.0370 (15)	0.0645 (19)	0.0588 (18)	−0.0071 (14)	0.0183 (14)	−0.0115 (16)
C5	0.0482 (17)	0.0492 (18)	0.0612 (18)	0.0026 (13)	0.0267 (15)	0.0045 (15)
C6	0.0366 (14)	0.0536 (17)	0.0415 (15)	0.0052 (12)	0.0155 (12)	0.0037 (13)
C7	0.0409 (13)	0.0369 (14)	0.0438 (15)	0.0050 (11)	0.0204 (12)	0.0045 (11)
C8	0.0445 (16)	0.0543 (18)	0.0625 (19)	−0.0063 (13)	0.0159 (14)	−0.0042 (15)
C9	0.070 (2)	0.0468 (18)	0.0565 (19)	−0.0033 (15)	0.0132 (17)	−0.0089 (14)
C10	0.084 (2)	0.0563 (18)	0.0522 (19)	0.0169 (17)	0.0323 (18)	−0.0001 (15)
C11	0.0528 (17)	0.066 (2)	0.061 (2)	0.0148 (15)	0.0330 (16)	0.0026 (16)
C12	0.0381 (14)	0.0541 (17)	0.0512 (16)	0.0005 (12)	0.0192 (13)	−0.0029 (13)

### Geometric parameters (Å, °)

**Table:**

Cl1—C2	1.731 (3)	C7—C8	1.508 (4)
Cl2—C5	1.738 (3)	C7—C12	1.510 (4)
S1—O1	1.427 (2)	C7—H7	0.9800
S1—O2	1.431 (2)	C8—C9	1.532 (5)
S1—N1	1.585 (2)	C8—H8A	0.9700
S1—C1	1.781 (3)	C8—H8B	0.9700
N1—C7	1.469 (3)	C9—C10	1.521 (5)
N1—H1n	0.88 (2)	C9—H9A	0.9700
C1—C6	1.384 (4)	C9—H9B	0.9700
C1—C2	1.392 (3)	C10—C11	1.507 (5)
C2—C3	1.370 (4)	C10—H10A	0.9700
C3—C4	1.371 (5)	C10—H10B	0.9700
C3—H3	0.9300	C11—C12	1.531 (4)
C4—C5	1.362 (4)	C11—H11A	0.9700
C4—H4	0.9300	C11—H11B	0.9700
C5—C6	1.379 (4)	C12—H12A	0.9700
C6—H6	0.9300	C12—H12B	0.9700
			
O1—S1—O2	120.23 (14)	C12—C7—H7	108.2
O1—S1—N1	108.69 (13)	C7—C8—C9	111.0 (2)
O2—S1—N1	106.64 (13)	C7—C8—H8A	109.4
O1—S1—C1	105.20 (13)	C9—C8—H8A	109.4
O2—S1—C1	106.27 (13)	C7—C8—H8B	109.4
N1—S1—C1	109.51 (13)	C9—C8—H8B	109.4
C7—N1—S1	124.32 (19)	H8A—C8—H8B	108.0
C7—N1—H1N	117 (2)	C10—C9—C8	111.3 (3)
S1—N1—H1N	117 (2)	C10—C9—H9A	109.4
C6—C1—C2	119.0 (2)	C8—C9—H9A	109.4
C6—C1—S1	117.89 (19)	C10—C9—H9B	109.4
C2—C1—S1	123.1 (2)	C8—C9—H9B	109.4
C3—C2—C1	120.4 (3)	H9A—C9—H9B	108.0
C3—C2—Cl1	118.2 (2)	C11—C10—C9	110.5 (3)
C1—C2—Cl1	121.3 (2)	C11—C10—H10A	109.6
C2—C3—C4	120.5 (3)	C9—C10—H10A	109.6
C2—C3—H3	119.7	C11—C10—H10B	109.6
C4—C3—H3	119.7	C9—C10—H10B	109.6
C5—C4—C3	119.1 (3)	H10A—C10—H10B	108.1
C5—C4—H4	120.4	C10—C11—C12	111.5 (3)
C3—C4—H4	120.4	C10—C11—H11A	109.3
C4—C5—C6	121.9 (3)	C12—C11—H11A	109.3
C4—C5—Cl2	119.5 (2)	C10—C11—H11B	109.3
C6—C5—Cl2	118.6 (2)	C12—C11—H11B	109.3
C5—C6—C1	119.1 (3)	H11A—C11—H11B	108.0
C5—C6—H6	120.5	C7—C12—C11	111.3 (3)
C1—C6—H6	120.5	C7—C12—H12A	109.4
N1—C7—C8	111.7 (2)	C11—C12—H12A	109.4
N1—C7—C12	109.0 (2)	C7—C12—H12B	109.4
C8—C7—C12	111.5 (2)	C11—C12—H12B	109.4
N1—C7—H7	108.2	H12A—C12—H12B	108.0
C8—C7—H7	108.2		
			
O1—S1—N1—C7	34.9 (3)	C3—C4—C5—C6	−1.0 (5)
O2—S1—N1—C7	165.9 (2)	C3—C4—C5—Cl2	179.6 (2)
C1—S1—N1—C7	−79.5 (2)	C4—C5—C6—C1	0.4 (4)
O1—S1—C1—C6	7.6 (2)	Cl2—C5—C6—C1	179.8 (2)
O2—S1—C1—C6	−120.9 (2)	C2—C1—C6—C5	0.1 (4)
N1—S1—C1—C6	124.3 (2)	S1—C1—C6—C5	178.1 (2)
O1—S1—C1—C2	−174.5 (2)	S1—N1—C7—C8	110.4 (3)
O2—S1—C1—C2	57.0 (3)	S1—N1—C7—C12	−126.0 (2)
N1—S1—C1—C2	−57.8 (2)	N1—C7—C8—C9	177.4 (3)
C6—C1—C2—C3	0.2 (4)	C12—C7—C8—C9	55.2 (4)
S1—C1—C2—C3	−177.7 (2)	C7—C8—C9—C10	−55.8 (4)
C6—C1—C2—Cl1	−179.3 (2)	C8—C9—C10—C11	56.1 (4)
S1—C1—C2—Cl1	2.8 (3)	C9—C10—C11—C12	−55.8 (4)
C1—C2—C3—C4	−0.9 (4)	N1—C7—C12—C11	−178.7 (2)
Cl1—C2—C3—C4	178.6 (2)	C8—C7—C12—C11	−55.0 (3)
C2—C3—C4—C5	1.3 (5)	C10—C11—C12—C7	55.5 (3)

### Hydrogen-bond geometry (Å, °)

**Table:**

*D*—H···*A*	*D*—H	H···*A*	*D*···*A*	*D*—H···*A*
N1—H1n···O2^i^	0.88 (2)	2.08 (2)	2.914 (3)	157 (2)
C4—H4···O1^ii^	0.93	2.60	3.246 (4)	127

Symmetry codes: (i) *x*, −*y*+2, *z*−1/2; (ii) *x*−1/2, −*y*+3/2, *z*−1/2.
